# BtpB, a novel *Brucella* TIR-containing effector protein with immune modulatory functions

**DOI:** 10.3389/fcimb.2013.00028

**Published:** 2013-07-08

**Authors:** Suzana P. Salcedo, María I. Marchesini, Clara Degos, Matthieu Terwagne, Kristine Von Bargen, Hubert Lepidi, Claudia K. Herrmann, Thais L. Santos Lacerda, Paul R. C. Imbert, Philippe Pierre, Lena Alexopoulou, Jean-Jacques Letesson, Diego J. Comerci, Jean-Pierre Gorvel

**Affiliations:** ^1^Aix-Marseille Univ UM 2, Centre d'Immunologie de Marseille-LuminyMarseille, France; ^2^INSERM U 1104Marseille, France; ^3^CNRS UMR 7280Marseille, France; ^4^Bases Moléculaires et Structurales des Systèmes Infectieux, CNRS UMR 5086, Institute of Biology and Chemistry of Proteins, Université Lyon 1Lyon, France; ^5^Instituto de Investigaciones Biotecnológicas Dr. Rodolfo A. Ugalde (IIB-INTECH), Universidad Nacional de San Martín, Consejo Nacional de Investigaciones Científicas y Técnicas, San MartínBuenos Aires, Argentina; ^6^URBM, NARILIS, University of Namur (FUNDP)Namur, Belgium; ^7^Laboratoire d'anatomie pathologique-neuropathologique, Aix-Marseille UniversitéMarseille, France

**Keywords:** *Brucella*, TIR domain, Btp1/BtpA, TLR, DC, NF-κB

## Abstract

Several bacterial pathogens have TIR domain-containing proteins that contribute to their pathogenesis. We identified a second TIR-containing protein in *Brucella* spp. that we have designated BtpB. We show it is a potent inhibitor of TLR signaling, probably via MyD88. BtpB is a novel *Brucella* effector that is translocated into host cells and interferes with activation of dendritic cells. *In vivo* mouse studies revealed that BtpB is contributing to virulence and control of local inflammatory responses with relevance in the establishment of chronic brucellosis. Together, our results show that BtpB is a novel *Brucella* effector that plays a major role in the modulation of host innate immune response during infection.

## Introduction

Innate immune recognition of microbial components is critical for the onset of an appropriate immune response against invading pathogens. Key contributors include the toll-like receptor (TLR)/IL-1R superfamily characterized by the presence of a conserved region designated TIR domain located in the cytosolic part of each TLR. The TIR domain is critical for protein-protein interactions between TLRs with the corresponding TIR-containing adaptors, which couple downstream protein kinases. This signaling cascade ultimately leads to activation of specific transcription factors such as nuclear factor-κB (NF-κB) and production of inflammatory mediators. Although a variety of TLR receptors have been described, in humans the most relevant for recognition of bacterial molecules are TLR2, TLR4, TLR5, and TLR9.

In addition to TLRs and their adaptors, TIR domains are present in plant resistance proteins that mediate hypersensitive responses to pathogens, as well as in a variety of bacteria, including species present in the human gut microbiota, soil bacteria and human pathogens (Spear et al., 2009; Zhang et al., 2011). Their evolutionary history is complex and their role in interaction with eukaryotic hosts remains mostly uncharacterized. Nevertheless, in a number of bacterial pathogens, bacterial TIR-containing proteins have been implicated in virulence or control of cellular responses. *Salmonella enterica* serovar Enteritidis TlpA is capable of reducing NF-κB activation by TLR4, IL-1R and MyD88-dependent pathways and to contribute to control of IL-1β secretion during infection (Newman et al., 2006). In the case of uropathogenic *E. coli* CFT073, the TIR-containing protein TcpC is able to interfere with TLR4 and TLR2 signaling by targeting MyD88 (Cirl et al., 2008) but also to inhibit TRIF- and IL-6/IL-1 dependent pathways (Yadav et al., 2010). During infection, TcpC is implicated in the control of secretion of TNF-α and IL-6 and *tcpC* mutants show a defect in intracellular replication in a mouse model of pyelonephritis. The *Yersinia pestis* TIR-containing protein YpTdp interacts with MyD88 to reduce IL-1β- and LPS-dependent signaling and to contribute to modulation of cytokine secretion during infection (Spear et al., 2012).

In the case of *Brucella* spp. two groups independently reported on the role of a TIR domain containing protein in control of TLR signaling (Cirl et al., 2008; Salcedo et al., 2008). Naming of this protein as Btp1 or TcpB, respectively, by two distinct laboratories has led to some confusion in the literature and has been misinterpreted by some as two independent proteins. Since neither Btp1 nor TcpB conforms to the international guidelines for bacterial nomenclature we will hereafter designate Btp1/TcpB as BtpA.

BtpA is present in *B. abortus* 2308 (BAB1_0279), *B. abortus* 9-941 (BruAb1_0274) and *B. melitensis* 16 M (BMEI1674) but is absent from *B. suis* 1330. Cirl et al. described that ectopically expressed BtpA cloned from *B. melitensis* 16 M is able to interfere with TLR4 and TLR2 signaling by directly interacting with MyD88. Several reports have proposed that BtpA targets the adaptor protein MAL/TIRAP (Radhakrishnan et al., 2009; Sengupta et al., 2010). Direct comparison of the *in vitro* interaction between BtpA and either MyD88 or TIRAP shows a stronger interaction with MyD88 (Chaudhary et al., 2011). BtpA has been shown to bind phosphoinositides at the plasma membrane (Radhakrishnan et al., 2009) but also to induce ubiquitination of TIRAP (Sengupta et al., 2010). In accordance to its modulation of TLR function, previous work from our laboratory described the role of the BtpA from *B. abortus* in the control of dendritic cell (DC) activation during infection (Salcedo et al., 2008). Purified BtpA was also shown to inhibit CD8^+^ T cell-mediated killing suggesting it may also control adaptive immune responses (Durward et al., 2012).

Here we present a novel *Brucella* effector with a TIR domain that we designated as BtpB. We show that BtpB efficiently inhibits TLR signaling and contributes to control of DC activation. Together, *Brucella* TIR-containing proteins BtpA and BtpB modulate host inflammatory responses during infection.

## Results

### Identification of a second *Brucella* TIR domain-containing protein

**Figure 1:**
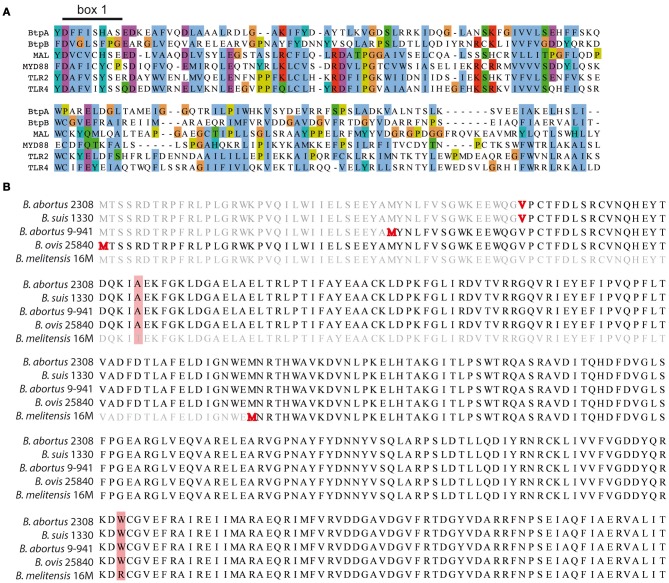
**Identification of BtpB. (A)** Identification of BtpB as bacterial member of TLR/IL-1R (TIR) family. Comparison of the predicted amino acid sequences of the TIR domain of BtpB with BtpA and the human members of the TIR family: MAL, MyD88, TLR2 and TLR4. The alignment was constructed with T-Coffee::advanced server from EMBnet (http://www.ch.embnet.org) and coloring scheme corresponds to standard ClustalX in which each residue in the alignment is assigned a color if the amino acid profile at each position meets a minimum criteria specific for the residue type. Box 1 corresponds to the signature sequence of the TLR family. **(B)** Alignment of BtpB amino acid sequences for *B. abortus* 2308 (BAB1_0756), *B. suis* 1330 (BR0735), *B. abortus* 9-941 (BruAb1_0752) and *B. melitensis* 16M (BME1216). The annotated starting codons (Methionine/Valine) are highlighted in red. Amino acid differences are shaded in red.

Analysis of the *Brucella* genome revealed the presence of a second TIR domain-containing protein (BAB1_0756) that we have designated BtpB (Figure 1A). We choose to continue with the Btp nomenclature to avoid any confusion with the *tcpB* gene necessary for conjugative transfer in *Clostridium perfringens* (Parsons et al., 2007). Search for conserved domains in BtpB revealed the presence of a C terminal TIR domain (aa 144-256) that belongs to the Pfam family TIR_2 (E value 1.9 *e*^−11^), a family of bacterial Toll-like receptors. TIR domains share conserved motifs called box 1 (F/Y-DAFISY), box2 (GYKLC-RD-PG) and box 3 (W residue surrounded by basic amino acids). Sequence comparison of BtpB TIR domain with the human TIR-containing proteins MAL, MyD88, TLR2 and TLR4 showed sequence similarity and conservation of box 1, essential for signaling (Rana et al., 2013) (Figure 1A).

Unlike BtpA, BtpB is present in all sequenced *Brucella* strains, including *B. suis* 1330 (BR0735), *B. abortus* 2308 (BAB1_0756), *B. abortus* 9-941 (BruAb1_0752) and *B. melitensis* 16 M (BME1216). Alignment of the *btpB* sequences derived from different *Brucella* strains revealed 4 different annotations for the start codon (highlighted in red in Figure 1B). Analysis of the −18 to +18 nucleotides around the ATG/GTG (Kolaskar and Reddy, 1985) predicted as the most likely start codon the second methionine highlighted in Figure 1B. This open reading frame has been annotated for *B. abortus* 9-941 and encodes a 292 amino acid protein, BtpB (1-292). The additional annotated start codons include the first highlighted methionine, the valine (GTG) and the *B. melitensis* methionine resulting in proteins of either 325, 277 or 178 amino acids. None of them scored high enough to be considered as likely start codons. Comparison of all *Brucella* sequences available revealed only one BtpB (1-178), in *B. melitensis* 16 M, whereas the majority correspond to BtpB (1-292). In consequence, we decided to use in this study the BtpB (1-292).

**Figure 2:**
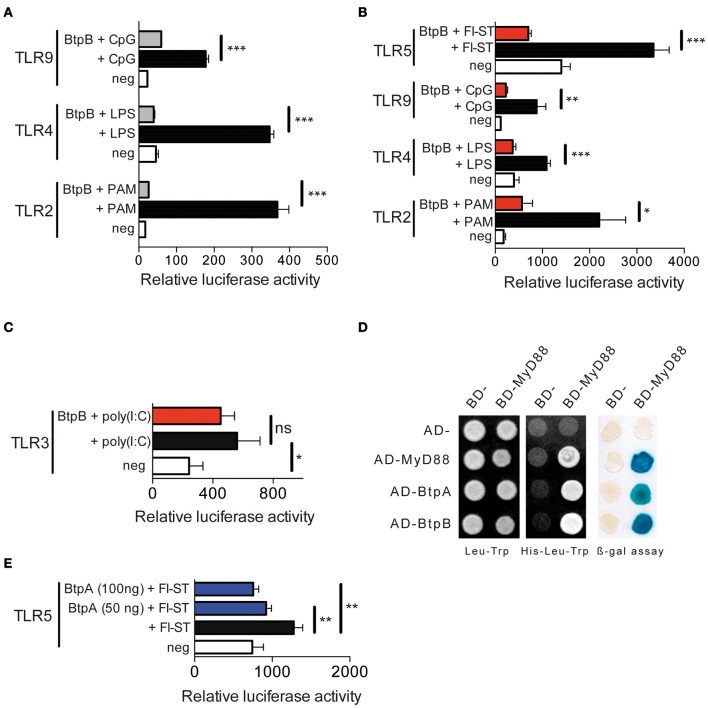
**BtpB interferes with TLR signaling. (A)** HEK293 cells were transiently transfected for 24 h with the luciferase reporter vector and either TLR2, TLR4 and TLR9, in the presence or the absence of the 178 amino acid BtpB (50 ng). Cells were then stimulated with the appropriate ligand (PAM, LPS and CpG) for 6 h before measurement of luciferase activity. White bars correspond to negative control, black bars to cells stimulated with the appropriate ligand and grey bars to cells transfected with BtpB and stimulated with the ligand. Data represent the means ± standard errors of relative luciferase activity obtained from triplicates of a representative experiment. **(B)** Luciferase activity in the presence or absence of the BtpB (1-292) (red bars). TLR5 was also included and stimulated with Flagellin from *S. typhimurium* (Fl-ST) and **(C)** TLR3 following stimulation with poly(I:C). **(D)** Yeast containing Gal4 BD- and Gal4 AD-fusion proteins were selected on synthetic medium lacking leucine (Leu) and tryptophan (Trp) (left panel). Protein interactions were identified on synthetic medium lacking histidine (His) and supplemented with 20 mM 3AT (middle panel). Growth on this medium indicates interaction between fusion proteins. The blue yeast colonies observed in the β-galactosidase expression filter assay indicate interaction between the fusion proteins (right panel). BD and AD indicate empty vectors and were used as negative controls, while MyD88 homodimerization was used as positive control. **(E)** Luciferase activity in cells transfected with TLR5 in the absence or presence of 100 ng and 50 ng of BtpA (275 aa). *P* ≤ 0.001 are denoted with ^***^; *P* ≤ 0.01 are denoted with ^**^ and *P* between 0.01 and 0.05 are denoted with ^*^.

We first investigated the ability of BtpB to interfere with TLR signaling using an *in vitro* NF-κB-dependent luciferase reporter system. BtpB was able to inhibit TLR2, TLR4 and TLR9 signaling (Figure 2A) even more efficiently than BtpA (Salcedo et al., 2008). This inhibition was independent on the first 114 amino acids as both BtpB (1-178) (Figure 2A), as well as, BtpB (1-292) (Figure 2B) strongly inhibited TLR signaling. BtpB was also able to inhibit flagellin-induced TLR5 signaling (Figure 2B). These results suggest that BtpB may interfere with a common molecule of these TLR pathways, such as MyD88. Consistently, BtpB did not reduce TLR3-dependent signaling which does not involve the adaptor MyD88 (Figure 2C). In addition, we observed by directed yeast two-hybrid that BtpB was able to interact with MyD88 (Figure 2D). BtpA was also able to interact with MyD88 by yeast-two hybrid as previously shown by pull-down and protein-fragment complementation assays (Cirl et al., 2008; Chaudhary et al., 2011). Neither BtpA nor BtpB interacted with any of the TLR1 to TLR10 TIR domains nor with the adaptors TIRAP or TRAM. As BtpA was previously shown to reduce TLR2 and TLR4 signaling but not TLR9 (Cirl et al., 2008; Salcedo et al., 2008) we investigated its ability to interfere with TLR5, which is also dependent on MyD88. BtpA was able to significantly reduce TLR5 signaling, following stimulation with *S. typhimurium* flagellin (Figure 2E).

Overall, our results show that BtpB is a potent inhibitor of TLR signaling *in vitro*, which may result from binding to MyD88.

### BtpB is translocated into host cells

**Figure 3:**
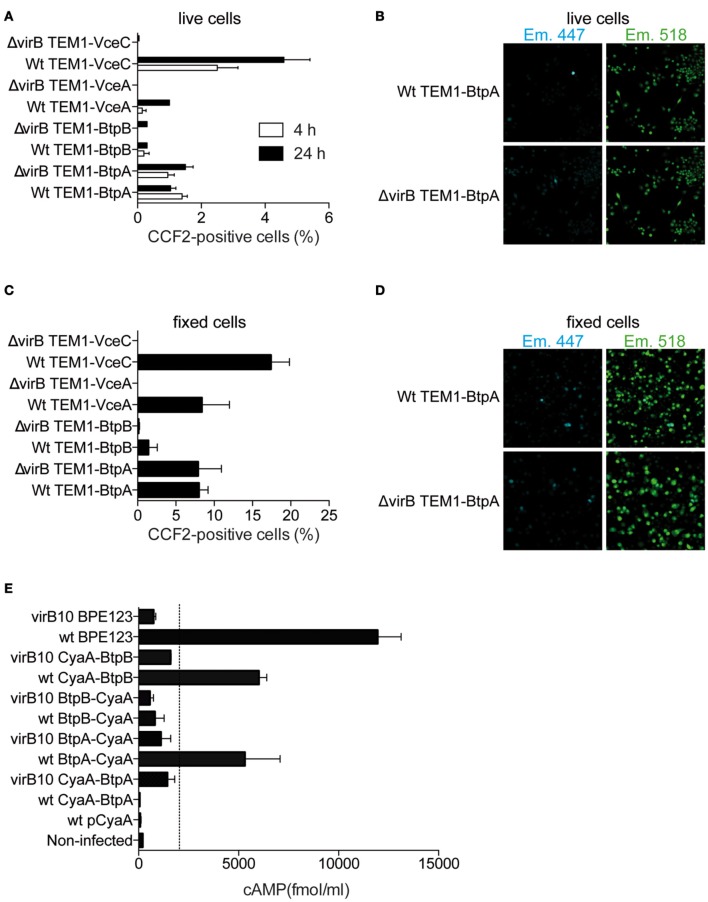
**BtpB is translocated into host cells during infection. (A)** RAW macrophages were infected with wild type (wt) or Δ*virB9 B. abortus* strains carrying N-terminal TEM-1 fused VceA, VceC, BtpA, and BtpB for 4 h and 24 h. Data represents the means ± standard errors of the percentage of cells with coumarin fluorescence from 5 independent experiments. **(B)** Representative confocal images of RAW cells infected with either wilt-type *B. abortus* (wt) or Δ *virB9* mutant carrying TEM-fused BtpA, at 24 h after inoculation. Appearance of blue cells is indicative of translocated TEM lactamase. **(C)** and **(D)** Analysis of TEM-1 translocation assay for fixed samples of VceA, VceC, BtpA, and BtpB 24 h after infection. **(E)** Intracellular cAMP levels in J774.A1 cells infected for 4 h with isogenic strains with a functional (wt) or non-functional VirB system (*virB10*) expressing Btp proteins fused to CyaA. Non-infected cells and a wild type strain expressing the CyaA domain alone (pCyaA) were included as negative controls. A wild type strain expressing BPE123-CyaA was included as a positive control. Means and SD are shown for one representative out of three independent experiments.

In order for BtpA and BtpB to target TLR pathways they would have to be exported across the bacterial membranes and the vacuolar membrane into the host cell cytosol. To test this hypothesis we analysed the translocation of BtpA and BtpB fused at their N-terminus with the TEM-1 β-lactamase during infection of RAW macrophage-like cells. This method has been successfully used to establish translocation of several *Brucella* effectors, namely VceA, VceC and RicA (de Jong et al., 2008; de Barsy et al., 2011) and is traditionally carried out in live cells. RAW cells were used in order to achieve high rates of infection. VceC and VceA were included as positive controls. We could detect BtpA translocation into host cells at 4 h and 24 h after inoculation (Figures 3A,B). Translocated BtpB was detected in less than 0.5% of infected cells at 4 h and did not significantly increase at 24 h. In an attempt to try to enhance the sensitivity of this assay, we carried out the same experiments in fixed samples with observation of FRET within 15 min of fixation, which enhances the shift to 450 nm (Nothelfer et al., 2011). As in live cells, BtpA and to a lower extent BtpB were translocated into host cells at 24 h after infection (Figures 3C,D). Since the overall percentage of cells showing translocated effectors is very low with this assay, even following fixation, we analysed translocation of BtpA and BtpB fused to the adenylate cyclase CyaA (Figure 3E). In addition, we used a constitutive promoter of *B. abortus bcsp31* gene to enhance expression, since this alternative approach was successfully used with the *Brucella* effector protein BPE123 (Marchesini et al., 2011), which was included as a positive control in our experiments. In this system, any value of cAMP bellow 1500–2000 fmol/ml corresponds to background (dotted line in Figure 3E) and is not indicative of translocation as determined after performing an exhaustive screening for the identification of *Brucella abortus* type IV secretion system (T4SS) substrates (Marchesini et al., 2011). We found that at 4 h after infection both BtpA and BtpB were translocated into J774.A1 macrophage-like cells (Figure 3E). Interestingly, translocation of BtpA fused with the CyaA seems to depend on the position of the tag as only BtpA with C-terminal CyaA was efficiently translocated into host cells at early stages of the infection. In contrast, for BtpB, the presence of the CyaA tag on the C-terminus reduced translocation (Figure 3E).

To determine if the translocation of BtpA and BtpB was dependent on the *Brucella* VirB T4SS, cells were infected with the *virB* mutant carrying either TEM- or CyaA-fused Btp proteins. We could not detect any differences between wild type and *virB* mutant using the TEM-1 β-lactamase assay (Figures 3A–D). In sharp contrast, translocation of BtpA-CyaA and CyaA-BtpB was clearly reduced in a *virB* genetic background, indicating that delivery of both proteins is dependent on the T4SS. We conclude that BtpA and BtpB are translocated into host cells and may constitute substrates for the VirB T4SS.

### BtpB replication within murine bone marrow-derived DCs

**Figure 4:**
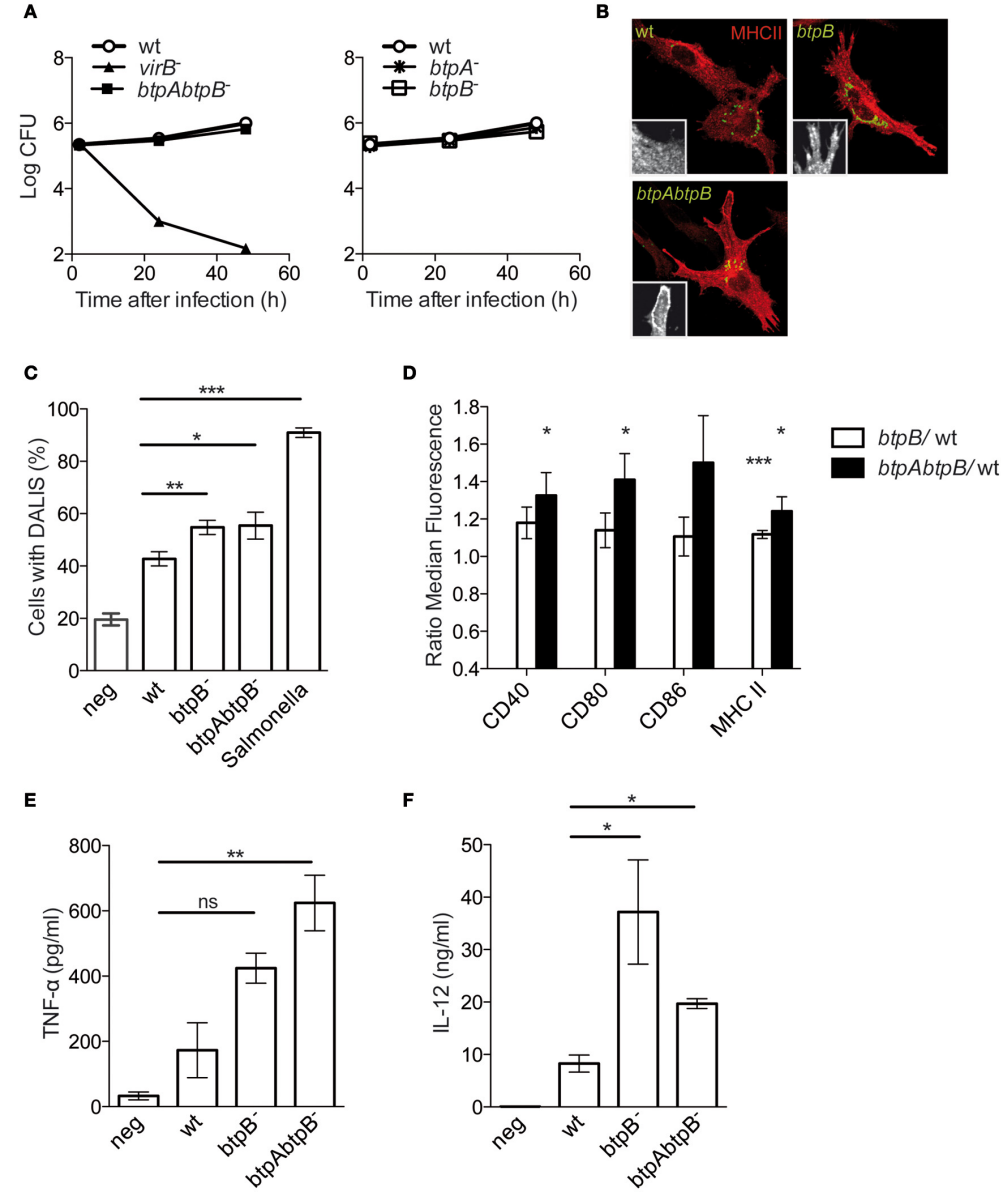
**Role of BtpB in control of DC activation. (A)** BMDCs infected with wild type *B. abortus* or the *btpAbtpB* mutant (left panel) and the single mutants (right panel) were lysed and intracellular CFUs enumerated at different times after inoculation. **(B)** Representative images of BMDCs infected with either the wild type, *btpB* or *btpAbtpB* mutants for 24 h. Cells were labeled for MHC class II (red) and surface expression is of a representative area is shown in zoom inlets. **(C)** Quantification of the percentage of DCs containing DALIS after 24 h of infection with wild type *B. abortus* (wt), *btpB*^−^ or *btpAbtpB* mutant. **(D)** Flow cytometry of the surface expression of MHC class II, CD40, CD80 and CD86 at 24 h post-infection. Data are normalized to wt values. **(E)** Analysis of TNF-α and **(F)** IL-12 (p40/p70) secretion measured by ELISA from the supernatant of DCs 24 h after inoculation. All the results correspond to the means ± standard errors of 4 independent experiments. *P* ≤ 0.001 are denoted with ^***^; *P* ≤ 0.01 are denoted with ^**^ and *P* between 0.01 and 0.05 are denoted with ^*^.

To determine the role of BtpB during infection we infected murine bone marrow-derived DCs with wild type *Brucella*, as well as, with a *btpA*, *btpB* or *btpAbtpB* mutant strains. No attenuation was observed as the *btpAbtpB* replicated to equivalent levels of the wild type *B. abortus* strain (Figure 4A). The survival curves for the single mutants overlap with that of the wild type (Figure 4A, right panel).

As previously described for BtpA, murine DCs infected with the *btpB* mutant showed higher level of MHC class II surface expression and higher percentage of formation of aggresome-like induced structures (DALIS) that transiently appear during the process of activation of these immune cells (Figures 4B,C) (Lelouard et al., 2002). However, there was no additive effect of depletion of both *btpA* and *btpB* as the *btpAbtpB* mutant did not show an increased phenotype compared to single mutant.

Flow cytometry analysis of infected cells did not reveal a statistically significant increase in CD40, CD80 and CD86 surface expression in DCs infected with *btpB* mutant when compared to the wild type at 8 h post-infection. At 24 h post-infection there was a significant increase in MHC class II surface expression in DCs infected with *btpB* mutant relative to those infected with the wild type (Figure 4D, white bars) consistent with our microscopy observations (Figure 4B). In the case of DCs infected with *btpAbtpB* mutant, CD40 and CD80 co-stimulation markers were up-regulated (Figure 4D, black bars).

In terms of cytokine secretion, BtpB did not seem to be involved in the control of TNF-α secretion during infection of murine DCs (Figure 4E). The increase in TNF-α secretion observed for *btpAbtpB* is probably due to a lack of BtpA, previously shown to be involved in the control of secretion of this cytokine (Salcedo et al., 2008). However, an increase in the level of total IL-12 (p40/p70) secreted during infection was observed in the case of the *btpB* mutant compared to the wild type 24 h after infection (Figure 4F). The difference between *btpB* and *btpAbtpB* mutants is not significant. These results suggest that BtpB is contributing to the control of the inflammatory response induced in infected DCs *in vitro.*

### BtpB controls NF-κB translocation in DCs

**Figure 5:**
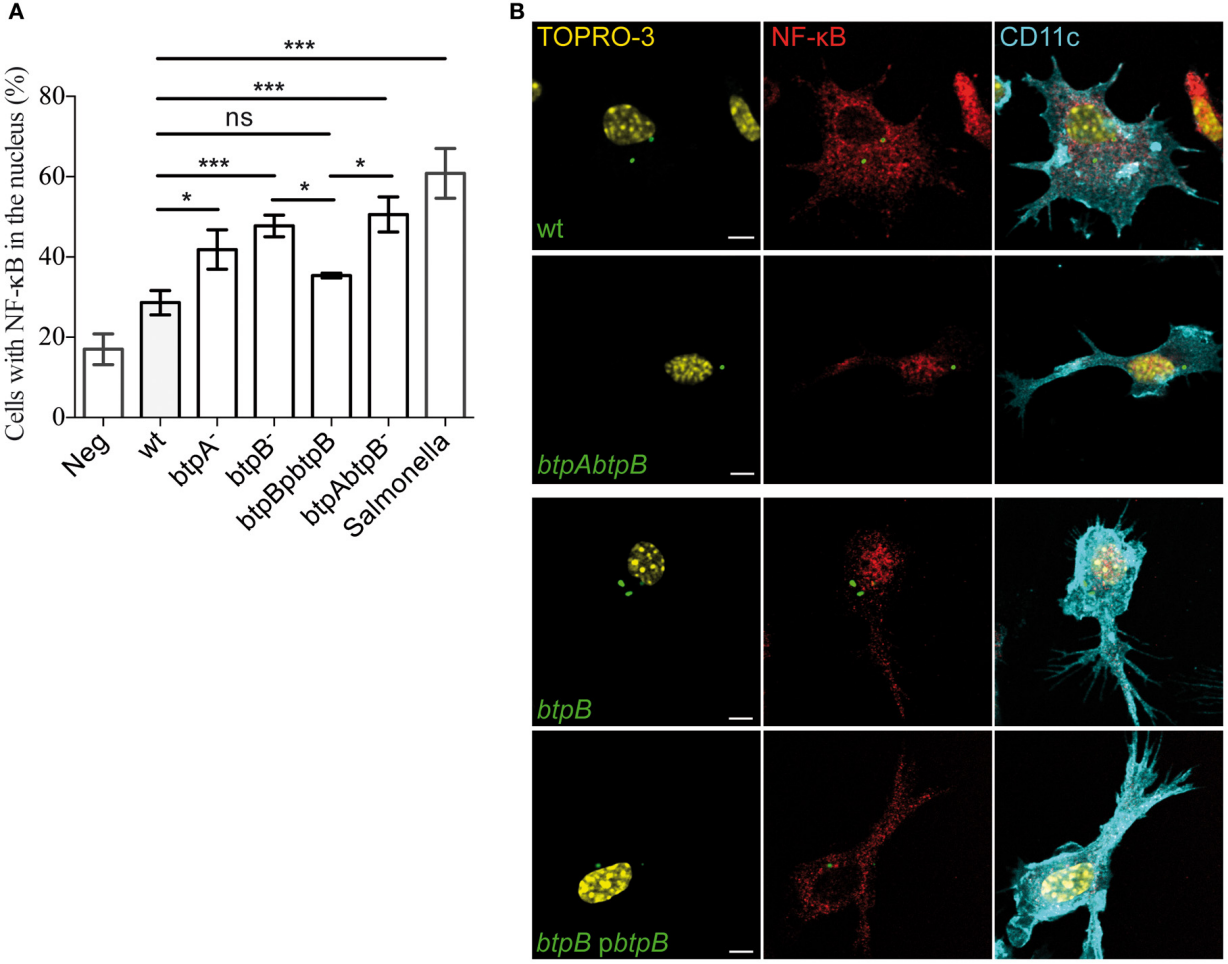
**Modulation of NF-κB translocation to the nucleus during *Brucella* infection.** Bone marrow-derived DCs were infected with wild type (wt) *B. abortus*, *btpA, btpB* and *btpAbtpB* mutants as well as *btpB* mutant carrying the complementing plasmid (p*btpB*) for 2 h and processed for immunofluorescence confocal microscopy. Cells were labeled for CD11c (cyan) and p65 NF-κB (red). Bacteria were labeled with anti-LPS antibody followed by FITC secondary and nuclei with TOPRO3. *Salmonella* infected cells were used as a positive control. **(A)** Data corresponds to means ± standard errors of 4 independent experiments. **(B)** Representative images obtained by confocal microscopy are shown for DCs infected with wild type, *btpB* mutant, *btpB*p*btpB* complemented strain and *btpAbtpB* mutant. Scale bars correspond to 5 μm. *P* ≤ 0.001 are denoted with ^***^; *P* ≤ 0.01 are denoted with ^**^ and *P* between 0.01 and 0.05 are denoted with ^*^.

In order to analyse the effect of the BtpB effector on the early stages of DC activation, translocation of NF-κB was monitored by immunofluorescence microscopy during the course of the infection. As early as 2 h post-infection, bone marrow-derived DCs infected with the *btpB* mutant showed an increased translocation of NF-κB into the nucleus compared to those infected with wild type *B. abortus* (Figure 5). The *btpB* mutant phenotype was rescued by expression of BtpB from a plasmid confirming the role of BtpB in the control of NF-κB translocation into the nucleus.

These results confirm that BtpB has an effect on the induction of inflammatory responses during *Brucella* infection.

### Role of BtpB in the mouse model of brucellosis

**Figure 6:**
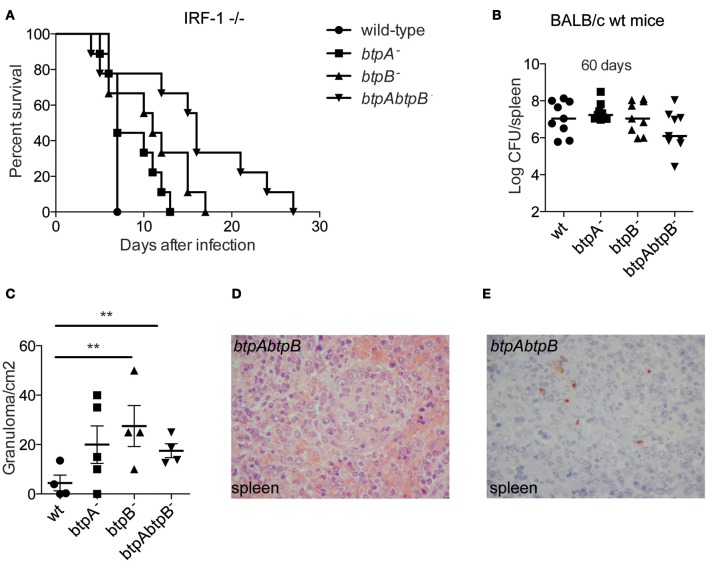
**Role of BtpB during *Brucella* infection in the mouse model of brucellosis. (A)** Susceptibility of IRF-1^−/−^ to *B. abortus* 2308 (wt), *btpA*^−^, *btpB*^−^ and *btpAbtpB* mutant (n = 9 per group). Infected mice were monitored daily for survival. Mice infected with *btpB* and *tpAbtpB*^−^ survived longer than wild type *Brucella* infected mice (*P* = 0.0433 and *P* = 0.0152, respectively). **(B)** Persistence of *B. abortus* 2308 (wt), *btpA, btpB or btpAbtpB* mutants in spleens of wild type BALB/c infected mice at 60 days p.i. Each symbol represents an animal and the median values are marked by horizontal bold lines. **(C)** Analysis of granuloma formation in the spleens of wild type BALB/c mice infected for 60 days with wild type *B. abortus*, *btpA, btpB* or *btpAbtpB* mutants. Data represent means ± standard deviations of 4 or 5 mice. **(D)** Representative image from the spleen of a mouse infected with *btpAbtpB* mutant (hematoxylin-eosin, original magnification ×400). **(E)** Bacteria were revealed by immunostaining in the spleen of wild type BALB/c mice infected by *btpAbtpB* mutant of *B. abortus*. Macrophages present in inflammatory granulomas in the red pulp are packed with coarse immunopositive material (hemalun counterstain, original magnification ×400). *P* ≤ 0.01 are denoted with ^**^.

To further investigate the role of BtpB during infection we carried out *in vivo* studies. BtpA *B. melitensis* mutants were previously shown to have enhanced survival in immuno-compromised Interferon Regulatory Factor-1 (IRF-1)^−/−^ mice (Radhakrishnan et al., 2009) inoculated intra-peritoneally (i.p.), a lethality model that has been used for studying *Brucella* virulence (Ko et al., 2002). We therefore, inoculated IRF^−/−^ i.p. with either *btpA*, *btpB* or *btpAbtpB B. abortus* mutants. Although the *btpB* mutant had no defect in intracellular replication in cultured cells *in vitro*, it showed an attenuation phenotype in IRF-1^−/−^ mice. Mice infected with the *btpB* mutant survived longer than those infected with the wild type *B. abortus* and synergistic effect was observed for the double *btpAbtpB* mutant (Figure 6A, *P* < 0.005), despite equivalent bacterial CFU counts in the spleen at each sampling date after infection (median of 1.05 × 10^8^ CFU/spleen for wild type versus 6.5 × 10^7^ CFU/spleen for the *btpAbtpB* mutant). To better study the role of BtpB in brucellosis *in vivo*, we inoculated wild type BALB/c mice i.p. and enumerated the bacterial load at 30, 60, 90, and 130 days post-infection. No significant differences in bacterial CFU counts between the wild type *Brucella* and the *btp* mutants were observed at different stages of the infection (30, 60, 90, and 130 days). Data at 60 days post-infection is shown as an example (Figure 6B). We then performed histological examination of spleens obtained from wild type BALB/c mice infected with the *btpA, btpB*, *btpAbtpB* mutants or the wild type *B. abortus* strains to quantify granuloma formation, which usually reflects the host's ability to develop a protective immune response. No granuloma was seen in the spleen of non-infected mice. In infected mice, granulomas were detected in splenic red pulp. A significantly higher number of granulomas was observed following *btpB* and *btpAbtpB* infection (after 60 days) compared to the wild type *Brucella* (Figure 6C). Inflammatory granulomas showed a similar organization in all populations of infected mice and were composed mainly of macrophages and a few lymphocytes (Figure 6D). Bacteria were detected by immunohistochemistry in the spleen of mice infected with wild type *B. abortus* or with the *btpAbtpB* mutant (Figure 6E). They were seen as coarse granular immune-positive material associated with cells, which had the morphology of macrophages.

These results are consistent with a role for BtpB in the control of inflammatory response during *Brucella* infection *in vivo*.

## Discussion

Previous work from our laboratory demonstrated a role for BtpA in control of DC activation. Here we show that *Brucella* contains a second TIR-domain protein called BtpB that is translocated into host cells and which participates in the control of the inflammatory response during *Brucella* infection. *In vitro*, BtpB is a potent inhibitor of TLR2, TLR4, TLR5 and TLR9. Together with BtpA, BtpB contributes to the control of DC activation during infection.

Using the TEM-1 lactamase and CyaA assays we were able to detect BtpA and BtpB translocated into the cytosol as early as 4 h after infection of RAW and J774 macrophages. This is an essential step to enable BtpA and BtpB to cross the bacterial and vacuolar membranes to reach their host cellular targets during infection. It would be interesting to localize the translocated proteins during infection. We have not been able to detect neither 2HA- nor 3FLAG-tagged BtpA and BtpB by immunofluorescence microscopy. It is possible that the amounts of translocated BtpA and BtpB are too low or perhaps these proteins are quickly degraded once they reach the host cytosol. It is important to note that using the TEM-1 lactamase assay alone we were unable to detect translocation of BtpB and any differences between wild type and *virB* mutant in BtpA translocation. It is possible that the low sensitivity of the TEM-1 lactamase compared to the CyaA assay makes this methodology inappropriate to assess VirB dependency in the case of effectors translocated at low levels. We conclude from our results that BtpA and BtpB are likely substrates of the VirB T4SS.

In this study, we found that *B. abortus* lacking *btpA* and *btpAbtpB* mutants showed an increased survival time in the IRF-1 ^−/−^ mouse model, highlighting the importance of these TIR-containing proteins in virulence. Similar results were obtained for *B. melitensis* lacking BtpA, which is defective in systemic spread at early stages of infection (Radhakrishnan et al., 2009). However, the use of such a severely immune-compromised mouse model hampers detailed analysis of the role of these proteins in control of inflammatory responses during infection. Therefore, we proceeded with our *in vivo* studies using immune-competent mice. We found that absence of BtpA and/or BtpB leads to increased granuloma formation in wild type mice, probably restricting bacterial dissemination as a consequence of the inability of the mutants to modulate the inflammatory response.

Infection of DCs with *B. abortus* lacking BtpB revealed that this effector protein is contributing to the modulation of the inflammatory response during infection. Interestingly, significant differences were observed between BtpA and BtpB. For example, BtpA had an impact on TNF-α secretion (Salcedo et al., 2008) but not BtpB, which affected surface expression of MHC class II and co-stimulatory molecules that we had not previously seen with BtpA. These differences may be due to different kinetics of translocation and time of action of each effector or perhaps the kinetics of the cellular processes affected. These differences could also be explained by specific targeting of host pathways. Interestingly, translocation of VceC results in enhanced pro-inflammatory responses as a result of the induction of the unfolded protein response by this T4SS effector (de Jong et al., 2012). This suggests that VirB effectors can have opposing effects, resulting in either activation of host immune responses or specific inhibition of inflammatory pathways. These differences may represent host cell or tissue specificity or simply reflect different stages of disease. It is now crucial to undertake a more global analysis of the specific contribution of these effectors during infection and a better characterization of host immune responses elicited *in vivo.* It is also possible that some of the phenotypes observed with effectors are simply an indirect or secondary effect of their action on eukaryotic cells during infection. Defining at the molecular level the effector cellular targets and analysing their contribution during infection will hopefully shed some light on these issues.

The host interacting partner of BtpA remains controversial. BtpA has been shown to induce degradation of phosphorylated TIRAP by enhancing its poly-ubiquitination (Sengupta et al., 2010) and to efficiently block TIRAP-induced NF-κB activation (Radhakrishnan et al., 2009). Together, these studies present TIRAP as the main target of BtpA whereas other groups have shown a direct interaction with MyD88 (Cirl et al., 2008; Chaudhary et al., 2011). Although comparison of the ability of BtpA to interact with TIRAP and MyD88 revealed a stronger binding to MyD88 (Chaudhary et al., 2011), surprisingly this interaction was dependent on the Death Domain of MyD88 and not the TIR domain. By yeast-two hybrid we found that both BtpA and BtpB can interact with MyD88. In the case of BtpB this result could explain its ability to block TLRs that are dependent on MyD88 signaling but not TLR3, which is dependent on the adaptor TRIF. Although inhibition of TLR2 and TLR4 by BtpA has been described, we did not detect any inhibition of TLR9 (Salcedo et al., 2008), which would be expected if BtpA was blocking MyD88. It is possible that inhibition of TLR9 by BtpA requires higher levels of expression of BtpA and could not be detected with our assay. Consistently, BtpA interfered with TLR5 signaling which is dependent on MyD88. Further work is now required to understand the molecular mechanism by which BtpA controls TLR activation, which may involve interaction and/or competition with both MyD88 and TIRAP.

In addition to control of inflammatory responses, BtpA has been shown to interact with phosphoinositides at the plasma membrane and modulate microtubule dynamics (Radhakrishnan et al., 2009, 2011). Ectopically expressed BtpA localizes to microtubules. These could constitute important activities that may also have a consequence on control of the inflammatory response, for example by misplacing specific adaptor molecules within the cell. In addition, these data indicate that BtpA may have additional eukaryotic targets yet to be identified. It will be interesting to evaluate during infection the contribution of these different functions of BtpA described *in vitro* and determine if they are dependent on the TIR domain or if other domains are contributing to assigning multiple functions to this effector. Our results strongly implicate BtpB in the control of host inflammatory responses during *Brucella* infection. However, it is possible BtpB has additional functions as it has been described for BtpA. We are currently determining if multiple pathways are targeted by BtpB to better understand the role of this novel effector during *Brucella* infection.

## Materials and methods

### Bacterial strains

The bacterial strains used in this study were *S. enterica* serovar Typhimurium strain 12023, smooth virulent *B. abortus* strain 2308 (Pizarro-Cerdá et al., 1998) and the isogenic mutants *virB9*^−^ (Celli et al., 2005)*, virB10*^−^ (Sieira et al., 2000), *btpA*^−^ (Salcedo et al., 2008), *btpB*^−^ (this study) and *btpA*^−^ *btpB*^−^ (this study). In the case of *Brucella*, green fluorescent protein (GFP)-expressing derivatives contain a pBBR1MCS-2 (Kovach et al., 1995) derivative expressing the *gfp-mut3* gene under the control of the lac promoter. *Brucella* strains were grown in tryptic soy broth (TSB; Sigma-Aldrich) and *Salmonella* in Luria Bertani (LB) medium. For infection, we inoculated 2 ml of media for 16 h at 37°C up to an optical density (OD_600 *nm*_) of approximately 2.0 (Celli et al., 2003). *Salmonella* strains were cultured 16 h at 37°C with aeration to obtain stationary phase cultures.

### Construction of btpB and btpAbtpB mutants

The *btpB* gene (BAB1_0756) was amplified from *B. abortus* genomic DNA using primers 5'-acgcgacctttccggctccctt-3′ and 5′-ttcggctagacaggaatgcatg-3′ and ligated to pGem-T-Easy vector (Promega) to generate pGem-TbtpB. The plasmid was linearized with EcoRV. Linearized pGem-btpB was ligated to a fragment containing a kanamycin resistance cassette to generate pGem-TbtpB::Kan. This plasmid was electroporated into *B. abortus* 2308 where it is incapable of autonomous replication. Homologous recombination events were selected using kanamycin resistance (50 μg/ml) and carbenicillin sensitivity (50 μg/ml) in tryptic soy agar plates. PCR and sequencing analyses showed that the *btpB* wild type gene was replaced by the disrupted one. The mutant strain obtained was called *btpB*^−^. *btpAbtpB*^−^ double mutant was obtained after electropration of pGem-TbtpB::Kan into *btpA*^−^ mutant (Salcedo et al., 2008). Homologous recombination events were selected as previously described for *btpB*^−^single mutant. PCR and sequencing analyses confirmed that *btpA* and *btpB* wild type genes were replaced by the disrupted ones in the double mutant strain.

### Construction of the btpB complemented strain

A DNA fragment coding for BtpB (325 aa) was amplified by PCR using primers 5′-atggatccgtggcgaatgaaccaatccgc-3′ and 5′-gcactagtctaggtgatgagggcgacgcg-3′. The PCR product was inserted by the flanking BamHI/SpeI sites (underlined) in the corresponding sites of pBBR1 MCS-4 (Kovach et al., 1995). The integrity of the construct was confirmed by sequence analysis. The plasmid was introduced into *btpB*^−^ mutant by biparental mating.

### Construction of tem-1 and CyaA fusions

DNA fragments coding for VceC, VceA, BtpA (1-275), and BtpB (1-292) were amplified by PCR, digested with XbaI and PstI and cloned into pFlagTEM1 (Raffatellu et al., 2005). Primers sequences with XbaI and PstI sites (underlined) are: VceC-Fw: 5′-tcctctagagaacgttcagagcgtccagaa-3′; VceC-Rv: 5′-aaactgcagctaattgcgggtttctcccttg-3′; VceA-Fw: 5′-tcctctagaaaaatcatcatcacggcagca-3′; VceA-Rv: 5′-aaactgcagctagttcttgggcgcgtggcc-3′; BtpA-Fw: 5′- tcctctagaagttcgtactcttctaatatt-3′; BtpA-Rv: 5′- aaactgcagtcagataagggaatgcagttc-3′; BtpB-Fw: 5′- tcctctagatacaatttatttgtttcgggc-3′; BtpB-Rv: 5′- aaactgcagctaggtgatgagggcgacgcg-3′. The integrity of all constructs was confirmed by sequence analysis. Plasmids were introduced into *B. abortus* 2308 or ΔvirB9/virB9^−^ by electroporation. pFlagTEM1 encodes a copy of TEM1 β-lactamase, in which the Sec-dependent signal sequence has been deleted and replaced with a 3 × FLAG tag at the N-terminus (Raffatellu et al., 2005). Expression of the fusion proteins in *Brucella* was confirmed by Western blot using a mouse anti-FLAG M2 antibody (Sigma-Aldrich). To generate plasmids coding for fusions to the N-terminus of CyaA, BamHI/SpeI DNA fragments coding for BtpA (1-275) and BtpB (1-292) were obtained by PCR amplification with primers carrying BamHI/SpeI sites and ligated into the corresponding sites of pCyaA (Marchesini et al., 2011). Primers sequences with BamHI and SpeI sites (underlined) are: BtpA-Fw: 5′-atggatccatgagttcgtactcttctaata-3′; BtpA-Rv: 5′-ggactagtgataagggaatgcagttcttt-3′; BtpB-Fw 5′- atggatccatgtacaatttatttgtttcgggc-3′; BtpB-Rv: 5′-ggactagtggtgatgagggcgacgcgctc-3. To generate plasmids coding for fusions to the C-terminus of CyaA, the genes coding for BtpA (1-275) and BtpB (1-292) with flanking XbaI and SacII sites (underlined) were amplified using primers BtpA-Fw: 5′-tatctagaatgagttcgtactcttctaatattg-3′/BtpA-Rv: 5′-tccccgcggtcagataagggaatgcagttc-3′ and BtpB-Fw: 5′-tatctagaatgtacaatttatttgtttcgggct-3′/BtpB-Rv: 5′-tccccgcggctaggtgatgagggcgacgcg-3′. The DNA fragment coding for CyaA was amplified with flanking BamHI and SpeI sites (underlined) using primers 5′-cgggatccatgcagcaatcgcatcaggct-3′ and 5′-cgactagtaaggctgtcatagccggaatcctggc-3′. DNA fragments coding for CyaA and BtpA or BtpB were ligated in the corresponding sites of pDK51 under *bcsp31* gene promoter as described in (Marchesini et al., 2011). The integrity of all constructs was confirmed by sequence analysis. Plasmids expressing CyaA fusion proteins were introduced in *B. abortus* strains by biparental mating. Expression of the fusion proteins in *Brucella* was confirmed by Western blot using a mouse serum raised against CyaA.

### Bacterial infection and replication assays

BMDCs were prepared from 6-8 week-old female C57BL/6 mice (Lelouard et al., 2002). Infections were performed at a multiplicity of infection of 30:1. Bacteria were centrifuged onto BMDCs at 400 g for 10 min at 4°C and then incubated for 30 min at 37°C with 5% CO_2_ atmosphere. Cells were gently washed twice with medium and then incubated for 1 h in medium supplemented with 100 μg/ml streptomycin to kill extracellular bacteria (or gentamicin for *Salmonella*). Thereafter, the antibiotic concentration was decreased to 20 μg/ml. Control samples were always performed by incubating cells with media only and following the exact same procedure for infection. To monitor bacterial intracellular survival, infected cells were lysed with 0.1% Triton X-100 in H_2_O and serial dilutions plated onto TSB agar to enumerated CFUs.

### Immunofluorescence microscopy NF-kB

Cells were fixed in 3% paraformaldehyde, pH 7.4, at room temperature for 20 min. Cells were then permeabilized for 10 min with 0.1% saponin in PBS, followed by a blocking for 1 h with 2% BSA in PBS. Primary antibodies were incubated for 1 h followed by 3 washes in PBS, 1 h incubation for secondary antibodies, 2 washes in PBS and 1 wash in water before mounting with Prolong Gold (Life technologies). Primary antibodies used: rabbit anti-p65 from Santa Cruz at 1/250, hamster anti-CD11c from BioLegend at 1/100 and cow anti-*Brucella* LPS antibody at 1/2000. Secondary antibodies used: goat anti-hamster Alexa 594, donkey anti-rabbit Cy3, goat anti-cow FITC, all from Jackson Immunoresearch. Nuclei were stained with TOPRO-3.

Samples were examined on a Leica SP5 laser scanning confocal microscope for image acquisition. Images of 1024 × 1024 pixels were then assembled using Adobe Photoshop 7.0. In all experiments we used an anti-CD11c antibody confirming analysis of DCs only. Quantification was always done by counting at least 100 cells in 4 independent experiments, for a total of at least 400 host cells analysed.

### Flow cytometry of infected cells

BMDCs were harvested 8 h or 24 h after infection and stained for 20 min at 4°C with anti CD11c APC-Cy7, anti CD40 Alexa 647, anti CD80 Pe-Cy5, anti CD86 FITC and anti MHC class II PE (all purchased at BioLegend). Cells were then washed once in 1% FCS in PBS and once in PBS. Cells were fixed for 20 min in 3% PFA at room temperature. At least 100,000 CD11c+ events were collected on flow cytometry using a FACS Canto II (Becton Dickinson) and analysis was done on FlowJo software (TreeStar).

### Tem translocation assay

RAW cells were seeded in a 96 well plates at 1 × 10^4^ cells/well overnight. Cells were then infected with an MOI of 500:1 by centrifugation at 4°C, 400 g for 5 min and 20 min at 37°C 5% CO_2_. Cells were then washed twice with DMEM and 200 μl of complete media, with gentamicin (50 μg/ml) and 1 mM of IPTG was added for 1 h. Media was replaced by 200 μl of complete DMEM, with gentamicin (10 μg/ml) and 1 mM of IPTG. At 4 or 24 h after infection cells were washed with 100 μl HBSS. 20 μl of CFF2 mix (as described by Life Technologies protocol) was then added to each well, and plate incubated for 1.5 h at room temperature in the dark. Cells were finally washed with 100 μl PBS and analysed immediately by microscopy. A total of 5000 cells were counted from 5 independent experiments in an automated manner using imageJ.

### CyaA assays

Translocation of BtpA and BtpB into host cells was assayed using the CyaA fusion approach. After infection of J774.A1 cells (MOI 250:1) for 4 h in 96-wells plates (10^5^ cells/well), cells were gently washed five times with PBS and lysed. Intracellular cAMP levels were determined by Direct cAMP Enzyme Immunoassay Kit (Sigma, CA200) as described by the manufacturer.

### Cytokine measurement

Sandwich enzyme-linked immunosorbent assays (ELISA) from ebioscience were used to detect IL-12 (p40/p70) and TNFα from supernatants of BMDCs infected with different *Brucella* strains.

### Luciferase activity assay

HEK 293 T cells were transiently transfected using Fugene (Roche) for 24 h, according to manufacturer's instructions, for a total of 0.4 μg of DNA consisting of 50 ng TLR plasmids, 200 ng of pBIIXLuc reporter plasmid, 5 ng of control Renilla luciferase (pRL-null, Promega) and 50 ng of myc-BtpA or myc-BtpB expression vectors. The total amount of DNA was kept constant by adding empty vector. Where indicated, cells were treated with *E. coli* LPS (1 μg/ml), Pam_2_CSK4 (100 ng/ml), CpG ODN1826 (1 μM), Flagellin Fl-ST (1 μg/ml) and poly(I:C) (25 μg/ml), all obtained from Invivogen, for 6 h and then cells were lysed and luciferase activity measured using Dual-Glo Luciferase Assay System (Promega). The BtpB constructs were obtained by cloning in the gateway (Life Technologies) entry vector and then cloned in pMyc. The following primers were used for BtpB (178 aa) ggggacaagtttgtacaaaaaagcaggcttcatgaatcgtacgcactgggcg and as reverse primer ggggaccactttgtacaagaaagctgggtcctaggtgatgagggcgacgcg. For BtpB (1-292) the forward primer was ggggacaagtttgtacaaaaaagcaggcttctacaatttatttgtttcgggc. The BtpA (1-275) primers were: ggggacaagtttgtacaaaaaagcaggcttcatgagttcgtactcttctaatatt and the reverse primer was ggggaccactttgtacaagaaagctgggtctcagataagggaatgcagttc.

### Yeast two-hybrid assay

The plasmids used for the Y2H interaction test were obtained by using the Gateway™ technique, except the pACT2 vector encoding human MyD88-Gal4 activation domain (AD) fusion that was provided by L. O'Neill. Briefly, human MyD88 and *B. melitensis* 16 M BtpA (BMEI1674) were amplified by PCR respectively from the pACT2-MyD88 vector and from genomic DNA with Gateway™ primers (GWMyD88F and GWMyD88R; GWbtpAF and GWbtpAR). PCR products were then separately cloned into the entry vector pDONR201 (Invitrogen Life-technologies) as previously described (Dricot et al., 2004). For btpB, the corresponding entry vector pDONR201-BMEI1216 from the ORFeome was used (Dricot et al., 2004). LR reactions were then performed as recommended by the manufacturer (Invitrogen Life-technologies) in order to clone MyD88 into pVV212 (Van Mullem et al., 2003) downstream of the Gal4 DNA-binding domain (BD), and BtpA and BtpB into pVV213 (Van Mullem et al., 2003) downstream of the Gal4 AD. Haploïd Saccharomyces cerevisiae strains Mav103 and Mav203 (Walhout and Vidal, 2001) were transformed with BD and AD fusion protein vectors respectively. Diploid yeasts carrying both plasmids were obtained by mating and selected on synthetic dextrose medium (SD) lacking leucine (leu) and tryptophan (trp) as previously described (Hallez et al., 2007). Protein interactions were assessed on medium lacking histidine (his) supplemented with 20 mM triaminotriazole (3AT). The β-galactosidase expression filter assay using the LacZ reporter gene was performed as described previously (Dozot et al., 2010). The primers used for two-hybrid constructs were: GWMyD88Fggggacaagtttgtacaaaaaagcaggctcgcgatggctgcaggaggtcccg ;GWMyD88Rggggaccactttgtacaagaaagctgggtaagggcagggacaaggccttg ;GWbtpAFggggacaagtttgtacaaaaaagcaggctcgatgagttcgtactcttctaat ;GWbtpARggggaccactttgtacaagaaagctgggtagataagggaatgcagttc.

### Mouse infection studies

Groups of 7- to 9-week-old female IRF 1^−/−^ or BALB/c mice were intraperitoneally inoculated with 10^6^ CFU of *B. abortus* strains in 0.2 ml PBS. The infected mice were housed in cages within a biosafety level 3 facility and IRF 1^−/−^ mice were monitored daily for survival. At the indicated times post-infection, spleens from infected mice were removed and homogenized in 2 ml of PBS. Tissue homogenates were serially diluted and plated in duplicate on TSA with the appropriate antibiotic. CFU were counted after 3–4 days of incubation at 37°C.

### Histological and immunohistological analysis

For each mouse, the spleen was removed, fixed with buffered formalin 4%, and embedded in paraffin. Serial sections (3 μm) of these specimens were obtained for routine hematoxylin-eosin and immunohistochemical investigations to assess the presence of granulomas and bacterial antigens, respectively.

Granulomas were defined as collections of ten or more macrophages within the organs. The inflammatory granulomas present in each tissue section of the spleens were counted during microscopic examination, and the total area of tissue sections was determined by quantitative image analysis as described previously (Stein et al., 2005). The results were expressed as the number of granulomas found per surface unit (i.e., square centimeters). Counts of granulomas were expressed as the mean ± the standard deviation per square centimeter and compared by using the Student *t* test. Immunohistochemical analysis was performed with a rabbit anti-*B. abortus* antibody used at a 1:1000 dilution with hemalun counterstain. The immunohistological procedure, in which an immunoperoxidase kit was used, has been described elsewhere (Leone et al., 2004). For each section, a negative control was performed with normal rabbit serum.

### Statistical analysis

Unpaired two-tailed Student's t test was carried out to determine the statistical differences between experimental data sets. *P* ≥ 0.05 were not considered significant; *P* ≤ 0.001 are denoted with ***; *P* ≤ 0.01 are denoted with ** and *P* between 0.01 and 0.05 are denoted with *. Statistical differences between IRF-1 ^−/−^ mice survival curves were determined with Mantel-Cox test.

### Conflict of interest statement

The authors declare that the research was conducted in the absence of any commercial or financial relationships that could be construed as a potential conflict of interest.
